# A Study of T Cell Tolerance to the Tumor-Associated Antigen MDM2: Cytokines Can Restore Antigen Responsiveness, but Not High Avidity T Cell Function

**DOI:** 10.1371/journal.pone.0000353

**Published:** 2007-04-04

**Authors:** Gavin M. Bendle, Shao-An Xue, Angelika Holler, Hans J. Stauss

**Affiliations:** Centre de Recherche Public-Santé, Luxembourg

## Abstract

**Background:**

Most tumor-associated antigens (TAA) currently used for immunotherapy of cancer are also expressed in normal tissues, which may induce tolerance and impair T cell-mediated immunity. However, there is limited information about how physiological expression in normal tissues alters the function of TAA-specific T cells.

**Methodology/Principal Findings:**

We used a T cell receptor transgenic model to study how MDM2 expression in normal tissues affects the function of T cells specific for this TAA that is found at high levels in many different types of tumors. We found that some MDM2-specific T cells escaped thymic deletion and persisted in the peripheral T cell pool. When stimulated with antigen, these T cells readily initiated cell division but failed to proliferate and expand, which was associated with a high rate of apoptosis. Both IL-2 and IL-15 efficiently rescued T cell survival and antigen-specific T cell proliferation, while IL-7 and IL-21 were ineffective. Antigen-stimulated T cells showed impaired expression of the effector molecules CD43, granzyme-B and IFN-γ, a defect that was completely restored when T cells were stimulated in the presence of IL-2. In contrast, IL-15 and IL-21 only restored the expression of CD43 and granzyme-B, but not IFN-γ production. Finally, peptide titration experiments with IL-2 rescued T cells indicated that they were of lower avidity than non-tolerant control T cells expressing the same TCR.

**Conclusions/Significance:**

These data indicate that cytokines can rescue the antigen-specific proliferation and effector function of MDM2-specific T cells, although this does not lead to the recovery of high avidity T cell function. This study sheds light on possible limitations of immunotherapy approaches that target widely expressed TAA, such as MDM2.

## Introduction

Mechanisms of both central and peripheral tolerance have evolved to silence T cells with specificity for self-antigen. Whilst these mechanisms of T cell tolerance reduce the risk of autoimmunity, they also represent an obstacle to successful T cell-based cancer immunotherapy, as most T cell-recognised tumor antigens are also expressed in some normal tissues [1]–[5]. A better understanding of the mechanism and the magnitude of tolerance to tumor-associated antigens (TAAs) will be essential for the rational design of TAA-specific immunotherapy strategies for the treatment of cancer. As the antigen expression pattern in normal tissues is likely to have a major impact on T cell tolerance, it is desirable to develop models that can provide information on how the physiological expression of TAAs in normal tissues affects the development and function of TAA-specific T cells.

In the past T cell receptor (TCR) transgenic models have been used successfully to provide insights into the mechanisms of central and peripheral tolerance. Antigen expression in the thymus results in clonal deletion of developing T cells, although deletion is sometimes incomplete as T cells can escape into the periphery [6], [7]. For antigens that are expressed outside the thymus, antigen encounter in the periphery may result in T cell deletion or the induction of a state of unresponsiveness, often referred to as anergy [8]–[11]. Identified mechanisms that can lead to unresponsiveness include down-regulation of the TCR and accessory molecules such as CD8 co-receptor, or the disruption of signalling pathways required for IL-2 production [6], [10], [12]–[14]. In addition to deletion and anergy, ignorance is a form of tolerance where the expression of antigens in certain immunologically privileged tissues is ignored by peripheral T cells [15]. Whilst ignorant tolerance is easily broken by immunization, deletion is irreversible, and strategies for the reversal of anergy may require some knowledge of the mechanism and the level of T cell unresponsiveness.

Recently, two transgenic mouse lines expressing TCRs specific for two defined TAAs have been developed in order to analyse how TAA expression in normal tissues affects T cell function. In one model, the TCR was specific for an epitope of gp100, a TAA that is over-expressed in most melanomas while its physiological expression is restricted to melanocytes [16]. In another model, the TCR was specific for an epitope in the gag protein of Friend Murine Leukaemia Virus (FMuLV), which is a major CTL target in FMuLV-transformed murine tumors [17]. In this case gag-transgenic mice were generated to target the expression of this model TAA to normal liver tissue. The immunological analysis in these two models revealed a relatively low level of tolerance against gp100, whilst T cell tolerance against gag was more profound. It is possible that these difference were due to differences in the tolerogenic properties of skin melanocytes versus the liver, although it is also possible that high level of transgene driven gag expression may have contributed to a more pronounced T cell tolerance. Here, we set out to assess tolerance to a TAA that is naturally expressed in most normal tissues.

MDM2 is over-expressed in a large number of human malignancies of haematological and epithelial origin [18]–[23]. The MDM2 oncogene functions by inactivating the p53 tumor suppressor protein [24], [25], and its over-expression in tumors was shown to contribute to the process of malignant transformation [18]–[23]. Using HLA-A2 transgenic mice and the allo-restricted strategy to circumvent tolerance to MDM2, we have previously demonstrated that CTL specific for an HLA-A2 presented peptide can kill a panel of human leukemia cell lines, while MDM2 expression levels in normal cells were too low to trigger CTL killing [26]. We also observed that CTL with this specificity were not detectable in HLA-A2 positive humans, which was compatible with the suggestion that tolerance mechanisms may have inactivated or deleted such CTL.

As the MDM2 expression pattern in normal tissues is similar in humans and mice, we generated TCR transgenic mice to study tolerance mechanisms against this TAA. Previously, we isolated from H2^d^ BALB/c mice high avidity allo-restricted CTL clones specific for an MDM2-derived peptide, pMDM100, presented by H2-K^b^ class I molecules [27], [28]. In this study, the TCR genes from the CTL were isolated and used to produce transgenic H2^d^ mice that did not present the TCR recognised pMDM100 peptide (referred to as Ag^neg^ mice), and H2^dxb^ F1 transgenic mice in which the peptide is presented by normal tissues (referred to as Ag^pos^ mice). These mice enabled us to determine how physiological MDM2 expression in normal tissues affected the development and function of T cells specific for this TAA.

We found that some MDM2-specific T cells escaped thymic deletion and accumulated in the periphery. When stimulated with antigen, these T cells initiated cell division but failed to expand and showed poor production of the T cell effector molecules CD43, granzyme-B and IFN-γ. IL-7 and IL-21 did not rescue T cell expansion, although IL-21 enhanced CD43 and granzyme-B expression, but not IFN-γ production. Both IL-2 and IL-15 restored robust T cell expansion, which correlated with a strong up-regulation of Bcl-2 expression. Whilst IL-15 only restored CD43 and granzyme-B production, IL-2 rescued expression of all effector markers including the production of IFN-γ. Therefore, in terms of expansion and effector differentiation the IL-2-rescued T cells seemed equivalent to non-tolerant control T cells expressing the same MDM2-specific TCR. However, peptide titration experiments revealed that triggering of rescued T cells required higher peptide concentrations than triggering of non-tolerant control T cells. Together, the data show that while expansion and effector differentiation can be efficiently restored by cytokines, the impaired avidity of the rescued T cells might compromise their anti-tumor activity.

## Materials and Methods

### Mice

To generate pMDM100 specific TCR transgenic mice the Vα5 and Vβ7 TCR chain genes from a high avidity allo-MHC-restricted pMDM100-specific CTL clone [27], [28] were cloned into a CD2-vector (courtesy of Dr D. Kioussis) in preparation for microinjections into (C57BL/6×CBA) F1 (H2^bxk^) oocytes to produce transgenic mice. The transgenic mice were produced in collaboration with Dr M. Mendelson (Columbia University, USA). The Ag^neg^ mice (transgenic H2^d^ mice) used in the experiments reported here were crossed back with BALB/c mice for at least five generations. The Ag^pos^ mice (transgenic H2^dxb^ F1 mice) used were generated by mating the Ag^neg^ mice with C57BL/6 mice. Mice were typed for pMDM100-specific TCR expression by isolation of mouse-tail genomic DNA followed by polymerase chain reaction (PCR) screening. The oligonucleotide primer sequences used in the PCR reaction were: the CD2-5× forward primer (Invitrogen, UK): 5′-AACCCAGCTTTCCCTGAAAGTG-3′; the Vβ7-PSP reverse primer (Invitrogen): 5′-GCAGAATCCAGAATCAGGGAG-3′ or the Vα5-PSP reverse primer (Invitrogen): 5′-CACTGTCTCCCTCCCGGACAC-3′.

### Peptides, Cytokines and Cell Lines

The synthetic peptide pMDM100 (ProImmune, UK) corresponds to amino acid 100-107 (YAMIYRNL) of the murine MDM2 protein. Synthetic peptide pSV9 (ProImmune, UK) corresponds to the amino acids (FAPGNYPAL) of the Sendai virus. In a previous study we have demonstrated that both pMDM100 and pSV-9 bind efficiently to H2-K^b^ MHC class I molecules [29]. Peptides were dissolved in PBS to give a concentration of 2mM and stored at −20°C. Human recombinant IL-2 (Roche), human recombinant IL-15 (R&D Systems), human recombinant IL-7 (R&D Systems) and murine recombinant IL-21 (R&D Systems) were added to selected T cell cultures. RMA-S cells (H2^b^) were derived from RMA cells by mutagenesis [30]. RMA-S cells are TAP deficient due to a point mutation in the TAP2 gene and express low levels of MHC class I molecules compared to RMA cells [29], [31]. MBL-2 cells (H2^b^) were derived from a Moloney murine leukaemia virus induced T-cell lymphoma [32].

### Flow cytometric analysis

Spleen and lymph nodes (inguinal and lumbar) were harvested from 6–8 week old Ag^neg^ mice and Ag^pos^ mice and single cell suspensions obtained by macerating tissues through a 40 µm nylon cell strainer. Samples were stained in PBS (1% FCS) with the appropriate dilution of the relevant mAbs. Propidium iodide staining cells were excluded from analysis. Samples were acquired on an LSR2 flow cytometer (BD Biosciences, USA) and analysed using FACS Diva software. The following mAbs were used for flow cytometric staining: rat-anti-mouse Vβ7 FITC (Serotec, UK), rat-anti-mouse CD4 FITC, rat-anti-mouse CD8α APC, rat-anti-mouse CD8α Cy-Chrome 5 (CY-5), rat-anti-mouse Vβ7 PE, hamster-anti-mouse CD69 PE, rat-anti-mouse CD25 PE, rat-anti-mouse CD62L-FITC, rat-anti-mouse CD44 PE, rat-anti-mouse CD43 activation-associated glycoform-PE (all BD Biosciences).

To assess apoptosis, CD8^+^ T cells were isolated from splenocytes using a CD8α isolation kit (Miltenyi Biotec, Germany) according to the manufacturer's protocol, stimulated as described, stained with annexin V and PI (both BD Biosciences) according to the manufacture's protocol and then analysed by flow cytometry as described previously.

For intracellular staining of Granzyme B and Bcl-2, T cells were permeabilized with BD cytofix/cytoperm kit (BD Biosciences) and stained with rat-anti-mouse Granzyme B PE mAb (eBioscience) or hamster-anti-mouse Bcl-2 FITC mAb (BD Biosciences). Specificity controls were performed using the appropriate isotype mAbs. Samples were analysed by flow cytometry as described previously.

### Proliferation Assays

Antigen specific T cell proliferation was assessed by directly visualising the division of cells using the fluorescent cytosolic dye, Carboxyfluorescein Diacetate Succinimidyl Ester (CFSE), or by the uptake of ^3^[H]-thymidine. For CSFE labelling of cells, splencoytes from the transgenic mice were incubated with 3 µM CFSE (Molecular Probes, UK) for 3 min at 37°C in 1ml of PBS. In each well of a 96 well-plate, 2×10^5^ CFSE-labelled splenocytes were stimulated with 1×10^5^ irradiated (80 Gy) temperature-induced RMA-S coated with pMDM100 peptide (10 µM) or an irrelevant control peptide, pSV9 (10 µM). The appropriate cultures were supplemented with 10 U/ml IL-2, 10 ng/ml IL-7, 50 ng/ml IL-15 or 50 ng/ml IL-21. Cells were harvested at the appropriate time point, stained with CD8α and TCR Vβ7 mAbs and subjected to CFSE profiling by flow cytometry on CD8^+^Vβ7^+^ T cells. The proliferative response of the CTL in response to limiting concentrations of antigen was assessed in ^3^[H]-thymidine incorporation assays. Triplicate cultures were plated for each different experimental condition. 5×10^4^ temperature induced irradiated (80 Gy) RMA-S cells were plated onto 96-well plates with wells containing graded concentrations of peptide in medium and were incubated for 1 hour at 37°C, prior to the addition of 2×10^5 ^transgenic splenocytes and 10 U/ml IL-2. Cells were incubated at 37°C in 5% CO_2_ for 2 days, pulsed with 0.5 µCi ^3^[H]-thymidine (Amersham, UK) and incubated for a further 24 hours. The cells were harvested using a 96-well plate harvester (Skatron Instruments, Norway) onto a filter mat and thymidine incorporation measured by liquid scintillation counting on a β-counter (Wallac, Finland).

### IFN-γ assays

To measure antigen specific IFN-γ production, splenocytes from the transgenic mice were stimulated, culture supernatant harvested and IFN-γ measured in an IFN-γ ELISA. To compensate for the lower numbers of CD8^+^Vβ7^+^ T cells in the spleens of Ag^pos^ mice, IFN-γ production was assessed in cultures containing four-fold excess of splenocytes from Ag^pos^ mice compared to splenocytes from Ag^neg^ mice. Triplicate cultures were plated for each different experimental condition on 96-well round-bottom plates. In each well 1×10^4^ splenocytes from Ag^neg^ mice or 4×10^4^ splenocytes from Ag^pos^ mice were stimulated with 1×10^4^ irradiated (80 Gy) temperature-induced RMA-S coated with pMDM100 peptide (10 µM) or an irrelevant control peptide, pSV9. In some experiments, peptides were added directly to the splenocytes of F1 mice. The appropriate cultures were supplemented with 10 U/ml IL-2, 10 ng/ml IL-7, 50 ng/ml IL-15 or 50 ng/ml IL-21. For peptide titration the temperature induced irradiated RMA-S cells were plated onto 96-well plates with wells containing graded concentrations of peptide in medium and were incubated for 1 hour at 37°C prior to adding the transgenic splenocytes. After 72 hours, 50 µl culture supernatant was harvested from each well and murine IFN-γ was measured by sandwich ELISA using anti-IFN-γ antibodies (BD Biosciences). The activity in experimental samples was ascertained using the standard curve of mean absorbance values (OD) versus the dilution of recombinant IFN-γ in the supernatant.

The functional activity of IL-2 rescued CTL was measured by intracellular IFN-γ staining. Intracellular IFN-γ stainings were performed as previously described[28]. Samples were analysed using a FACScalibur™ flow cytometer and CELLQuest™ software.

### IL-2 biosssays

To measure antigen specific IL-2 production, splenocytes from the transgenic mice were stimulated, culture supernatant harvested and IL-2 measured using IL-2 dependent CTLL cells. The stimulation phase of the IL-2 assay was performed exactly as the IFN-γ assay. After 72 hours, 50 µl culture supernatant was harvested, transferred to wells containing CTLL cells (5×10^3^) and incubated for 16–18 hours. The cells were pulsed with ^3^[H]-thymidine by adding 1 µCi ^3^[H]-thymidine to each well and incubating for a further 12 hours. The activity in experimental samples was ascertained using the standard curve of cpm versus the dilution of recombinant IL-2 in the supernatant.

### CTL assays

Cytotoxic activity was determined in 4-hour ^51^chromium-release assays against MBL-2 tumor cells and RMA-S cells coated with pMDM100 peptides or MHC class I-binding control peptides as described [33].

## Results

### Transgenic T cells are present in the periphery of Ag^pos^ mice

**Figure 1:**
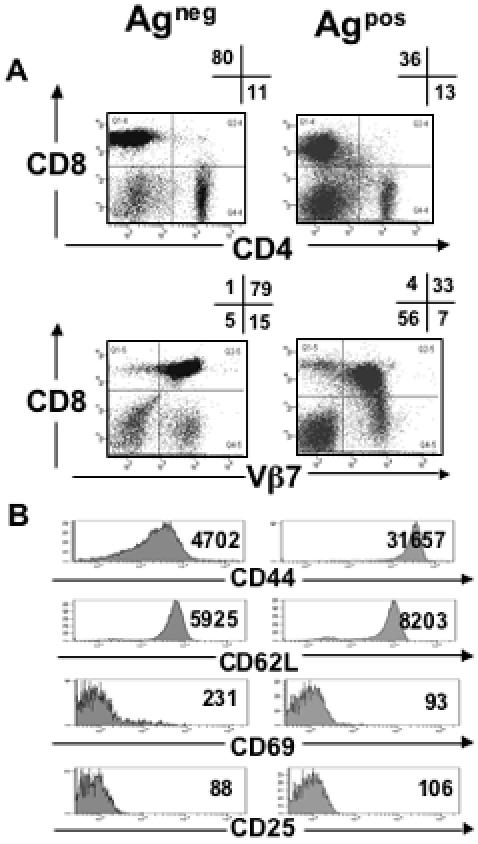
MDM2-specific CD8^+^ T cells are present in the periphery of Ag^pos^ mice and express reduced levels of TCR and CD8 but high levels of CD44. (A) Lymph nodes cells from 6–8 week old Ag^pos^ and Ag^neg^ mice were stained with antibodies against CD4, CD8 and Vβ7 and analysed by flow cytometry. Numbers in quadrants are percentage of live-gated lymphocytes. (B) Lymph node cells were stained with antibodies against CD8, Vβ7 and the activation markers CD44, CD62L, CD69 and CD25. Histograms display gated viable CD8^+^Vβ7^+^ cells. Numbers in histograms represent the specific MFI of the activation marker staining for the gated population. Data are representative of at least three independent experiments.

Monoclonal antibodies against CD4, CD8 and Vβ7 were used to identify cells expressing the transgenic TCR β chain (antibodies against the transgenic TCR α chain were not available). Although thymic deletion occurred in Ag^pos^ mice (data not shown), a substantial number of T cells was found in the periphery. The staining profile of the lymph node samples demonstrated a significant percentage of CD8^+^ T cells that expressed Vβ7 in the Ag^pos^ mice (Figure 1 A), and a similar profile was observed for splenic T cells (data not shown). The mean fluorescence intensity (MFI) of Vβ7 staining for the T cells of Ag^pos^ mice was 3075 compared to 5543 in the Ag^neg^ control mice (Figure 1A). Similarly, there was a decrease in the CD8 expression in T cells of Ag^pos^ mice compared to in the Ag^neg^ mice (MFI of CD8 staining: 13907 and 32526). The decreased CD8 expression in the Ag^pos^ mice was seen only on the CD8^+^ T cells that expressed the transgenic Vβ7 TCR but not on those that did not. These data demonstrated that the MDM2-specific T cells in the periphery of Ag^pos^ mice had down-regulated CD8 co-receptor and TCR expression.

Next the expression of the activation markers CD25, CD44, CD62L and CD69 was analysed on lymph node cells from Ag^pos^ and Ag^neg^ mice. The T cells from both sets of mice were CD25^lo^, CD69^lo^ and CD62L^hi^ (Figure 1B). While the T cells of Ag^neg^ mice expressed intermediate levels of CD44, which is associated with a naïve phenotype, the T cells of the Ag^pos^ mice were CD44^hi^, which is characteristic of antigen experienced T cells (Figure 1 B).

### T cells of Ag^pos^ mice display defects in antigen-driven expansion

**Figure 2:**
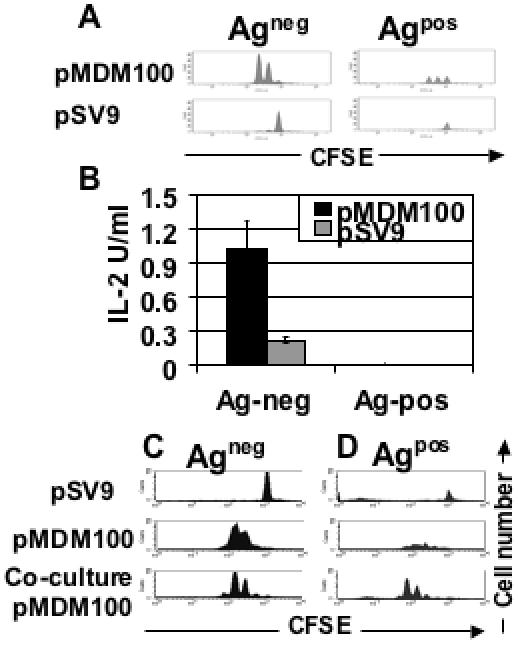
MDM2-specific T cells from Ag^pos^ mice display defects in antigen-driven expansion. (A) CFSE-labelled splenocytes from Ag^neg^ and Ag^pos^ mice were stimulated with RMA-S coated with pMDM100 peptide (10 µM) or pSV9 peptide (10 µM) for 48 hrs, stained with CD8 and Vβ7 mAbs and subjected to CFSE profiling by flow cytometry of gated CD8^+^Vβ7^+^ T cells. Data are representative of at least three independent experiments. (B) To measure antigen-specific IL-2 production, splenocytes from Ag^neg^ mice and Ag^pos^ mice were stimulated with RMA-S cells coated with pMDM100 peptide (100 µM) or a control peptide, pSV9 (100 µM). After 72 hours, 50 µl of culture supernatant was harvested and murine IL-2 was measured in a CTLL bioassay. Data represent the mean±SD of triplicate values and are representative of two independent experiments. (C) CFSE-labelled splenocytes from Ag^neg^ mice were stimulated with RMA-S cells coated with pMDM100 or SV9 control peptides for 72 hrs. In the bottom panel, CFSE-labelled T cells were stimulated for 72h with pMDM100 in the presence of unlabelled splenocytes from Ag^pos^ mice. (D) CFSE-labelled splenocytes from Ag^pos^ mice were stimulated with RMA-S cells coated with pMDM100 or SV9 control peptides for 72 hrs. In the bottom panel, CFSE-labelled T cells were stimulated for 72h with pMDM100 in the presence of unlabelled splenocytes from Ag^neg^ mice.

We then analysed antigen-specific proliferation of the T cells in the Ag^pos^ mice and Ag^neg^ mice. Splenocytes from the mice were CFSE labelled, stimulated *in vitro* with pMDM100-coated RMA-S cells followed by staining with antibodies against CD8 and Vβ7 to identify transgenic T cells. The CFSE labelled T cells from the Ag^neg^ mice divided in response to pMDM100 stimulation, with each cell division producing an increase in the number of viable T cells containing reduced levels of CFSE (Figure 2A). Whilst the T cells from the Ag^pos^ mice divided a similar number of times in response to antigen-specific stimulation, we failed to see an accumulation of the divided T cells (Figure 2 A). CFSE labelled T cells from the Ag^neg^ or Ag^pos^ mice stimulated with a H2-K^b^ binding control peptide, pSV9, did not proliferate in these CFSE assays (Figure 2A).

As IL-2 is a major autocrine growth factor produced by functionally competent CD8^+^ T cells, we explored if T cells of Ag^pos^ mice were able to express this cytokine. While non-tolerant control T cells of Ag^neg^ mice secreted IL-2 upon peptide stimulation, no IL-2 was detectable when the T cells of Ag^pos^ mice were stimulated (Figure 2B). Together, this suggested that the T cells displayed an anergic phenotype characterised by a lack of IL-2 production and an inability to expand upon peptide stimulation. Thus, we sought to determine whether the anergic T cells displayed regulatory activity and inhibited the proliferation of functionally competent T cells. To do this, CFSE labelled T cells from the Ag^neg^ mice were co-cultured with unlabelled tolerant T cells and stimulated with pMDM100 for 72 hours. CFSE-labelled non-tolerant T cells showed peptide-specific proliferation regardless of whether or not tolerant T cells were present in the cultures (Figure 2C). As expected, CFSE-labelled tolerant T cells showed defective expansion when stimulated with pMDM100 peptides. This defect was reversed when tolerant T cells were stimulated with peptide in the presence of non-tolerant T cells (Figure 2D), indicating that functionally competent T cells were able to restore the expansion of tolerant T cells. As non-tolerant T cells can produce IL-2 upon peptide stimulation (see above) it is possible that this cytokine may have triggered the expansion of the peptide-stimulated tolerant T cells. To explore this further, we tested directly whether IL-2 or other common gamma chain cytokines were able to restore the expansion of the tolerant T cells.

### IL-2 and IL-15 efficiently induce Bcl-2 expression and restore the expansion of tolerant T cells

**Figure 3:**
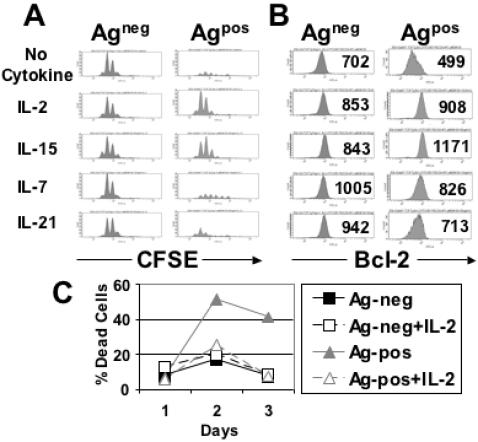
IL-2 and IL-15 efficiently induce Bcl-2 expression and restore the expansion of tolerant MDM2-specific CD8^+^ T cells. (A) CFSE-labelled splenocytes from Ag^neg^ and Ag^pos^ mice stimulated with RMA-S coated with pMDM100 peptide (10 µM) for 72 hrs in the presence of 10 U/ml IL-2, 10 ng/ml IL-7, 50 ng/ml IL-15 or 50 ng/ml IL-21, stained with CD8 and Vβ7 mAbs and subjected to CFSE profiling of gated CD8^+^Vβ7^+^ T cells. (B) Splenocytes from Ag^neg^ mice and Ag^pos^ mice were stimulated for 3 days *in vitro* with pMDM100-coated targets in the presence of 10 U/ml IL-2, 10 ng/ml IL-7, 50 ng/ml IL-15 or 50 ng/ml IL-21 and Bcl-2 expression levels were analysed by intracellular staining and flow cytometric analysis. Numbers in histograms represent the specific MFI of the gated CD8^+^Vβ7^+^ population. Following staining of the splenocytes with an appropriate isotype control mAb the MFI of the gated CD8^+^Vβ7^+^ population from the Ag^neg^ mice and Ag^pos^ mice was 98 and 97, respectively. Data in A and B are representative of three independent experiments. (C) CD8^+^ T cells isolated from the spleens of Ag^neg^ and Ag^pos^ mice were stimulated with RMA-S coated with pMDM100 peptide (10 µM) in the presence or absence of 10 U/ml IL-2 and cell death was determined on the indicated days by staining with annexin-V and propidium iodide and flow cytometric analysis.

CFSE-labelled T cells of Ag^pos^ mice were stimulated with peptide antigen in the presence of IL-2, IL-7, IL-15 or IL-21. FACS analysis revealed that peptide stimulation in the presence of IL-2 and IL-15 resulted in robust expansion of tolerant T cells (Figure 3A). The observed kinetics of cell division and cell accumulation was similar to that seen with peptide-stimulated non-tolerant control T cells of Ag^neg^ mice. In contrast, tolerant T cells stimulated with peptide and IL-7 or IL-21 showed a defect in the accumulation of divided cells similar to that seen after peptide stimulation without cytokines (Figure 3A). This indicated that IL-7 and IL-21 were unable to rescue the defective expansion of tolerant T cells.

Next, we explored whether IL-2, IL-7, IL-15 or IL-21 enhanced the expression of the anti-apoptotic molecule Bcl-2 in tolerant T cells. Peptide stimulation in the absence of cytokines revealed that the tolerant T cells expressed substantially less Bcl-2 than the non-tolerant control T cells (Figure 3B). However, stimulation in the presence of IL-15 and IL-2 resulted in higher levels of Bcl-2 expression in tolerant T cells compared to the control T cells. Although IL-7 and IL-21 were able to up-regulate Bcl-2 expression in tolerant T cells, the levels were lower than those seen after IL-2 and IL-15 stimulation. These data showed that the efficient Bcl-2 up-regulation seen with IL-2 and IL-15 correlated with the ability of these cytokines to rescue the expansion of tolerant T cells. Further experiments revealed that the defective expansion of tolerant T cells correlated with a high rate of apoptosis as revealed by annexin-V and propidium iodide staining (Figure 3C). When tolerant T cells were stimulated in the presence of IL-2 they showed a low level of apoptosis similar to that seen in non-tolerant control T cells. Thus, it is possible that up-regulation of Bcl-2 resulted in the suppression of apoptosis which promoted the expansion of the tolerant T cells. Alternatively, it is possible that IL-2 and IL-15 activated additional pathways required for T cell expansion, and that these pathways were not activated by IL-7 and IL-21.

### Rescue of defective effector differentiation of tolerant T cells

We then analysed the effect of the common gamma chain cytokines on the differentiation of tolerant lymphocytes into effector T cells. We measured IFN-γ secretion as this effector molecule is typically produced by CD8^+^ T cells. We also measured expression of the serine protease granzyme-B as it plays a key role in the granule-exocytosis pathway of CTL mediated target cell killing [34], [35]. Finally, we analysed the expression of the activation-associated glycoform of CD43 which has been shown to be up-regulated on effector T cells [36].

**Figure 4:**
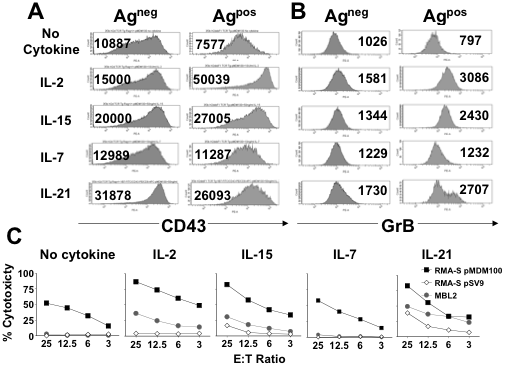
CTL from Ag^pos^ mice display defects at the level of cytotoxic effector function that can most effectively be rescued by IL-2. Splenocytes from Ag^neg^ mice and Ag^pos^ mice were stimulated for 3 days *in vitro* with pMDM100-coated targets in the presence of 10 U/ml IL-2, 10 ng/ml IL-7, 50 ng/ml IL-15 or 50 ng/ml IL-21. (A) CD43 expression levels on the stimulated T cells was analysed by surface staining and flow cytometric analysis. Numbers in histograms represent the specific MFI of the gated CD8^+^Vβ7^+^ population. (B) Granzyme B expression levels on the stimulated T cells was analysed by intracellular staining and flow cytometric analysis. Numbers in histograms represent the specific MFI of the gated CD8^+^Vβ7^+^ population. Following staining with an appropriate isotype control mAb the MFI of the gated CD8^+^Vβ7^+^ T cells from the Ag^neg^ mice and Ag^pos^ mice was 398 and 495, respectively. Data in A and B are representative of three independent experiments. (C) Cytolytic activity of stimulated T cells against MDM2-expressing MBL-2 tumor cells and RMA-S targets coated with pMDM100 peptide (10 µM) or a class I binding control peptide, pSV9 (10 µM). Data are representative of two independent experiments.

Upon peptide stimulation in the absence of cytokines, the tolerant T cells expressed lower levels of CD43 than non-tolerant control T cells. Stimulation of tolerant T cells in the presence of IL-7 resulted in a modest CD43 up-regulation. Both IL-15 and IL-21 triggered more substantial CD43 expression than IL-7, although the highest CD43 levels were induced by IL-2 (Figure 4A). Interestingly, IL-2 had only a modest effect on CD43 expression in non-tolerant control T cells, where IL-21 mediated the strongest CD43 up-regulation (Figure 4A).

The granzyme-B expression pattern of tolerant T cells was similar to that of CD43. Peptide stimulation in the absence of cytokines resulted in reduced granzyme-B expression in tolerant T cells compared to non-tolerant control T cells (Figure 4B). IL-2 was most effective in up-regulating granzyme-B expression, followed closely by IL-21 and IL-15, while IL-7 had only a modest effect. Finally, the granzyme-B expression pattern correlated with the cytotoxic activity of tolerant T cells. T cells rescued by IL-2, IL-15 and IL-21 killed peptide coated target cells and tumor cells more efficiently than T cells cultured with IL-7 or without cytokine supplementation (Figure 4C).

**Figure 5:**
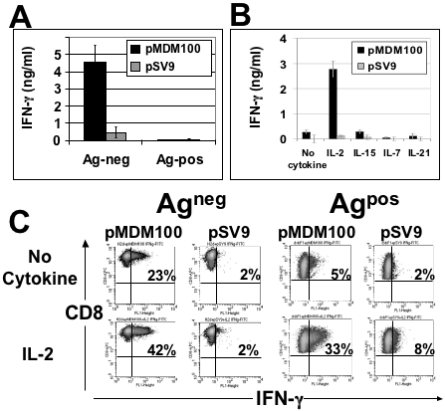
pMDM100-specific CD8^+^ T cells from Ag^pos^ mice display defects in IFN-γ effector function that can be restored by IL-2. (A) To measure antigen-specific IFN-γ production, splenocytes from Ag^pos^ mice and Ag^neg^ mice were stimulated with RMA-S cells coated with pMDM100 peptide (10 µM) or a control peptide, pSV9 (10 µM). After 72 hours, 50 µl of culture supernatant was harvested and IFN-γ was measured by ELISA. Data represent the mean±SD of triplicate values. (B) Antigen-specific IFN-γ production by splenocytes from Ag^pos^ mice stimulated with pMDM100 peptide or a control pSV9 peptide in the presence of 10 U/ml IL-2, 10 ng/ml IL-7, 50 ng/ml IL-15 or 50 ng/ml IL-21. After 72 hours, 50 µl of culture supernatant was harvested and IFN-γ was measured by ELISA. Data represent the mean±SD of triplicate values. Data in A and B are representative of three independent experiments. (C) Antigen-specific IFN-γ production upon secondary peptide stimulation of IL-2 rescued T cells was measured by intracellular staining for IFN-γ. T cells were re-stimulated with RMA-S cells coated with pMDM100 (10 µM) or an irrelevant control peptide, pSV9 (10 µM), for 5 hours in the presence or absence of 10 U/ml IL-2, followed by intracellular staining for IFN-γ. Comparable results were obtained when IFN-γ production by IL-2 rescued T cells was measured by ELISA in two independent experiments.

The analysis of the effector molecule IFN-γ revealed that tolerant T cells were unable to secrete IFN-γ after peptide stimulation in the absence of cytokine, whereas non-tolerant control T cells readily produced IFN-γ (Figure 5A). Whilst IL-2 efficiently restored IFN-γ secretion by tolerant T cells, the other common gamma cytokines IL-7, IL-15 and IL-21 were unable to promote IFN-γ production (Figure 5B).

Taken together, IL-2 was most effective in restoring all analysed defects of effector differentiation of tolerant T cells. The activity of IL-2 was similar to that of IL-15 and IL-21, except that the latter two cytokines only rescued CD43 and granzyme-B expression but were unable to restore IFN-γ production by tolerant T cells.

Subsequently we tested whether cytokine rescued T cells became functionally equivalent to non-tolerant T cells, or whether they remained cytokine dependent upon secondary peptide stimulation. Thus, tolerant T cells were first stimulated with peptide and IL-2 for 7 days, washed and re-stimulated with peptide in the absence or presence of IL-2. Secondary stimulation in the absence of cytokines resulted in poor IFN-γ production by tolerant T cells (5% positive T cells versus 2% background with the control peptide), whereas re-stimulation in the presence of IL-2 restored IFN-γ production by 33% of the T cells (Figure 5C). As expected, the secondary IFN-γ response of non-tolerant control T cells did not require the presence of IL-2. Peptide stimulation in the absence of IL-2 supplementation resulted in IFN-γ production by 23% of T cells, which increased to approximately 42% in the presence of IL-2. Together, these experiments indicated that IL-2 rescued tolerant T cells remained IL-2-dependent when re-challenged with peptide antigen.

### The cytokine rescued tolerant T cells display reduced functional avidity

**Figure 6:**
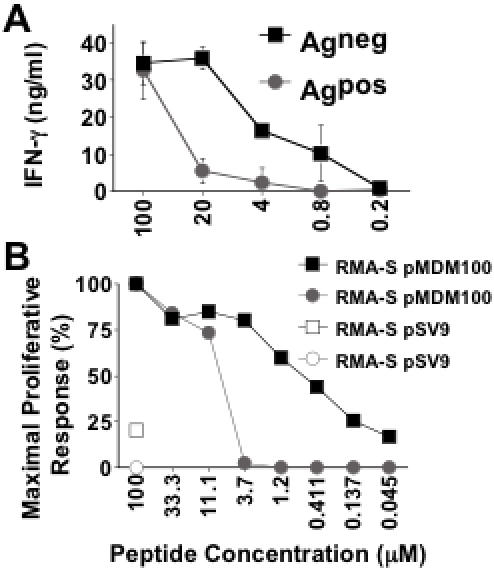
pMDM100-specific CD8^+^ T cells from Ag^pos^ mice display reduced functional avidity. (A) Splenocytes from Ag^neg^ mice and Ag^pos^ mice were stimulated with RMA-S cells coated with titrated concentrations of pMDM100 peptide or an irrelevant control peptide, pSV9, in the presence of 10 U/ml IL-2. After 72 hours, 50 µl of culture supernatant was harvested and IFN-γ was measured by ELISA. Data represent mean±SD of triplicate values. IFN-γ produced in response to RMA-S loaded with pSV9 was subtracted from all values. Data are representative of two independent experiments. (B) To measure antigen specific proliferation, splenocytes from Ag^neg^ mice (black squares) and Ag^pos^ mice (grey circles) were stimulated with RMA-S cells coated with titrated concentrations of pMDM100 peptide or an irrelevant control peptide, pSV9, in the presence of 10 U/ml IL-2 for 2 days, and then pulsed with 0.5 µCi ^3^[H]-thymidine for 24 hours. The maximal proliferative response by splenocytes from the Ag^neg^ and Ag^pos^ mice was 130480.2±9552.9 CPM and 56225.17±2794.9 CPM (triplicate values), respectively.

As IL-2 rescued tolerant T cells seemed functionally equivalent to the non-tolerant control T cells we assessed the avidity of these two T cell populations. IL-2 rescued T cells and control T cells were stimulated with decreasing concentrations of peptide followed by measurement of IFN-γ production. IL-2 rescued T cells required higher peptide concentrations to trigger IFN-γ production than non-tolerant control T cells (Figure 6A). Similarly, antigen-specific proliferation required higher peptide concentrations when IL-2 rescued tolerant T cells were compared with non-tolerant control T cells (Figure 6B). Therefore, despite expressing the same transgenic TCR, the cytokine rescued T cells were of lower functional avidity than the non-tolerant control T cells.

## Discussion

In this study we defined how physiological expression of the endogenous MDM2 protein shapes the response profile of T cells expressing an MDM2-specific TCR. The peripheral T cells responded to MDM2 peptides by initiating cell division, but the cells were unable to expand which correlated with a block in IL-2 production. This resembled the phenotype of the CD8^+^ T cells from protein kinase C-θ (PKCθ)-deficient mice [37]. CD8^+^ T cells in PKCθ-deficient mice underwent antigen-induced proliferation but displayed impaired survival and therefore failed to accumulate. Exogenous IL-2 or culture with wild-type T cells could correct the defective expansion of the PKCθ-deficient T cells, which is similar to the observations with the tolerant T cells analysed in this study. Therefore, it is tempting to speculate that a defect in the PKCθ pathway may underlie the defective expansion of the MDM2-specific T cells from Ag^pos^ mice.

We found that IL-2 was able to rescue the expansion and effector differentiation, including IFN-γ production, of tolerant T cells. Surprisingly, IL-15 was unable to rescue IFN-γ production by the tolerant MDM2-specific T cells. As previous studies have demonstrated that IL-15 stimulation can promote IFN-γ production by murine and human T cells [38], [39], this pathway seems to be defective in the tolerant T cells studied here. The observation that IL-15 stimulation enhanced T cell expansion and expression of certain effector markers indicated that the tolerant T cells were responsive to IL-15, but retained a selective defect in the pathway leading to IFN-γ expression. Furthermore, the differential effects of IL-2 and IL-15 in tolerant T cells indicated that these two common gamma chain cytokines use distinct mechanisms to trigger the production of IFN-γ. Whilst previous studies have identified a transcriptional control mechanism by which IL-2 induced IFNγ expression [40], the mechanism of IL-15-mediated IFN-γ production is currently less clear.

IL-21 was unable to rescue the expansion of the tolerant T cells, but was able to up-regulate the expression of CD43 and granzyme-B. Like IL-15, IL-21 did not promote IFN-γ production of peptide-stimulated tolerant T cells. Recent studies have shown that IL-21 can work synergistically with IL-15 to promote expansion and effector differentiation of naïve T cells in mice and in humans [38], [41]. It will be interesting to study how combinations of common gamma chain cytokines affect the function of the MDM2-specific T cells. A major advantage of IL-15 and IL-21 is that they can maintain the expression of the T cell co-stimulatory molecule CD28 and lymphoid homing markers such as CD62L [38], which was found to improved the in vivo survival and anti-tumor activity of adoptively transferred T cells in murine model experiments [39], [41], [42].

Recent studies analysed T cell tolerance of transgenic T cells expressing TCRs specific for the murine homologue of the melanoma antigen gp100 and the gag protein of FMuLV [16], [17]. The gp100-specific T cells seemed little affected by antigen expression in normal melanocytes, as they were functionally competent when stimulated with peptides *ex vivo* [16]. In contrast, the peptide stimulation of the gag-specific T cells showed that they were unable to proliferate, although they developed cytotoxicity and produced IFN-γ. IL-2 was relatively inefficient in reversing the proliferative defect of the gag-specific CTL, while IL-15 was highly effective in this model [17], [43]. The analysis of MDM2-specific CTL showed that both IL-2 and IL-15 restored T cell proliferation and expansion, indicating differences in the mechanism of tolerance induced by gag and MDM2. It is likely that the tissue-specificity and level of antigen expression determined the level of tolerance in the three transgenic models. The gp100 expression in melanocytes had little effect on T cell function, MDM2 expression in most normal tissues induced tolerance that was readily reversed by IL-2, whilst the expression of gag as a transgene using the albumin promoter rendered tolerant T cells relatively unresponsive to IL-2. Together, these TCR transgenic models provide valuable information about how distinct expression patterns in normal tissues affects the state of tolerance of TAA-specific T cells. Furthermore, these models can be used to gain insights into possible strategies to reverse tolerance of TAA-specific CTL.

We found that IL-2 was effective in restoring the function of MDM2-specific T cells *ex vivo*, although this did not lead to a complete restoration of T cell avidity. A possible explanation for the lower avidity of rescued T cells is the reduced expression levels of the TCR and the CD8 co-receptor seen in the tolerant T cells. It is interesting to note that MDM2 presentation in Ag^pos^ mice was sufficient to trigger TCR down-regulation, although previous studies suggested that effector CTL did not recognise normal tissues. For example, high avidity effector CTL specific for the same MDM2 peptide analysed here, selectively killed MDM2 expressing tumors but not normal cells [27], [28]. These observations suggested, that the antigen threshold required for the induction of tolerance in the transgenic T cells was lower that the threshold required for the triggering of cytotoxic effector CTL. However, it is possible that effector CTL remain susceptible to the tolerogenic signals that diminish the function of the tolerant T cells. We observed that adoptive transfer of MDM2-specific effector CTL into tumor-bearing resulted in the induction of T cell unresponsiveness [28]. This unresponsiveness was not due to tumor induced immune suppression, but was also seen after CTL transfer into tumor free animals. This is compatible with the idea that antigen expression in normal tissues can induce tolerance in adoptively transferred CTL, even when antigen levels are insufficient to trigger effector function. Thus, it is likely that adoptive T cell therapy needs to be combined with strategies that can protect transferred T cells from the induction of unresponsiveness. One of the beneficial effects of IL-2, which is typically administered in combination with adoptive T cell transfer [16], [43], [44], may relate to its ability to prevent anergy induction in transferred T cells.
